# Qingfei Tongluo Mixture Attenuates Bleomycin-Induced Pulmonary Inflammation and Fibrosis through mTOR-Dependent Autophagy in Rats

**DOI:** 10.1155/2024/5573353

**Published:** 2024-02-08

**Authors:** Shuyu Ge, Zhenghong Guo, Ting Xiao, Pingping Sun, Bo Yang, Yin Ying

**Affiliations:** ^1^Department of Pharmacy, Zhejiang Academy of Traditional Chinese Medicine, Tongde Hospital of Zhejiang Province, Hangzhou, China; ^2^College of Pharmacy, Guizhou University of Traditional Chinese Medicine, Guiyang 550025, China; ^3^The State Key Laboratory of Functions and Applications of Medicinal Plants, School of Pharmaceutical Sciences, Guizhou Medical University, Guiyang 550031, China

## Abstract

As an interstitial fibrosis disease characterized by diffuse alveolitis and structural alveolar disorders, idiopathic pulmonary fibrosis (IPF) has high lethality but lacks limited therapeutic drugs. A hospital preparation used for the treatment of viral pneumonia, Qingfei Tongluo mixture (QFTL), is rumored to have protective effects against inflammatory and respiratory disease. This study aims to confirm whether it has a therapeutic effect on bleomycin-induced IPF in rats and to elucidate its mechanism of action. Male SD rats were randomly divided into the following groups: control, model, CQ + QFTL (84 mg/kg chloroquine (CQ) + 3.64 g/kg QFTL), QFTL-L, M, H (3.64, 7.28, and 14.56 g/kg, respectively) and pirfenidone (PFD 420 mg/kg). After induction modeling and drug intervention, blood samples and lung tissue were collected for further detection. Body weight and lung coefficient were examined, combined with hematoxylin and eosin (H&E) and Masson staining to observe lung tissue lesions. The enzyme-linked immunosorbent assay (ELISA) and the hydroxyproline (HYP) assay kit were used to detect changes in proinflammatory factors (transforming growth factor-*β* (TGF-*β*), tumor necrosis factor-*α* (TNF-*α*), and interleukin-1*β* (IL-1*β*)) and HYP. Immunohistochemistry and Western blotting were performed to observe changes in proteins related to pulmonary fibrosis (*α*-smooth muscle actin (*α*-SMA) and matrix metalloproteinase 12 (MMP12)) and autophagy (P62 and mechanistic target of rapamycin (mTOR)). Treatment with QFTL significantly improved the adverse effects of bleomycin on body weight, lung coefficient, and pathological changes. Then, QFTL reduced bleomycin-induced increases in proinflammatory mediators and HYP. The expression changes of pulmonary fibrosis and autophagy marker proteins are attenuated by QFTL. Furthermore, the autophagy inhibitor CQ significantly reversed the downward trend in HYP levels and *α*-SMA protein expression, which QFTL improved in BLM-induced pulmonary fibrosis rats. In conclusion, QFTL could effectively attenuate bleomycin-induced inflammation and pulmonary fibrosis through mTOR-dependent autophagy in rats. Therefore, QFTL has the potential to be an alternative treatment for IPF in clinical practice.

## 1. Introduction

Idiopathic pulmonary fibrosis (IPF) is a pathological change in lung tissue characterized by diffuse inflammation and structural disorders of the alveoli, caused by the continuous action of multiple pathogenic factors. The progression of the disease can lead to the deposition of extracellular matrix (ECM) and epithelial transformation (EMT), which in turn forms lung fibrosis and eventually causes loss of function at the site of injury [1, 2]. The pathogenesis of IPF is complex, and various proinflammatory factors such as transforming growth factor-*β* (TGF-*β*), tumor necrosis factor-*α* (TNF-*α*), and interleukin-1*β* (IL-1*β*) have been confirmed to be involved in the two major stages of IPF development, namely inflammatory and fibrosis [3, 4]. Among them, TGF-*β* is considered the main factor that drives fibrosis. Overexpression of TGF-*β* induces fibrosis by activating both the TGF-*β*/Smad and noncanonical pathways, resulting in myofibroblast activation, excessive ECM production, and inhibition of ECM degradation [5]. In addition, TNF-*α* can also stimulate uncontrolled ECM protein deposition, leading to fibrosis and organ failure [6].

Autophagy is a dynamic process in which cells self-degrade and recover intracellular components, participating in various physiological processes to maintain the intracellular environmental balance. Whether autophagic flux is blocked or not is directly related to the progression of pulmonary fibrosis. A previous animal study showed that autophagy disruption played a crucial role in the regulation of bleomycin-induced pulmonary fibrosis formation [7]. In addition, insufficient autophagy was also observed in lung tissue from patients with pulmonary fibrosis, characterized by accumulation of p62, and reduced expression of LC3-II [8]. Consequently, improving autophagy was a well-known shielding mechanism to combat pulmonary fibrosis [9]. Recent research suggested that autophagy could promote the conversion of the myofibroblast phenotype and inhibit collagen deposition [10, 11]. It is noteworthy that, the mechanistic target of rapamycin (mTOR) played a negative role in autophagy by regulating autophagy-related proteins and lysosomal biosynthesis [12]. As a key factor in regulating autophagy, its increased expression enhanced the ability to regulate autophagy under stress conditions [13]. Therefore, inhibition of mTOR overactivation is a potential treatment method for IPF.

Bleomycin (BLM) is a first-line antitumor drug with side effects of pulmonary fibrosis. Its induced pulmonary fibrosis animal model was considered one of the most suitable, reproducible, and cost-effective experimental models [14].

Chloroquine (CQ) is a classic autophagy inhibitor that works primarily by inhibiting lysosomal acidification. It could neutralize the pH value of lysosomes to inhibit the activity of degrading enzymes, thus inhibiting the fusion of autophagosomes and lysosomes consistent with blocked autophagy [15]. Many studies have confirmed that CQ could achieve effective inhibition of autophagy in models of bleomycin-induced pulmonary fibrosis, and the addition of CQ was considered an effective strategy to verify whether drugs or molecules inhibit pulmonary fibrosis by regulating autophagy [16–18].

Pirfenidone (PFD) is currently one of the few drugs available for the treatment of IPF. It was discovered in 1976 but it only recently received approval in most countries. Although its efficacy was limited and even its exact mechanism of action was still unclear, PFD had shown efficacy in various animal models and was often used as a positive drug [19].

Qingfei Tongluo mixture (QFTL, approval No. Z20200045002) is a hospital preparation improved from classic formulas such as Qianwei Gan Tang and Liujunzi Tang. It is composed of 13 herbs, including Radix Pesudostellariae, Radix Adenophorae, Cortex Mori, Rhizoma Atractylodis Macrocephalae, Indigo Naturalis, Rhizoma Phragmitis, Radix Platycodi, Pheretima, Radix Salviae Miltiorrhizae, Semen Coicis, Semen Benincasae, Rhizoma Pinelliae, Fructus Amomi Villosi, and Radix Glycyrrhizae. Clinically, QFTL is used mainly to treat community-acquired pneumonia, epidemic pneumonia, and novel coronavirus pneumonia. Some herbs in QFTL, such as Rhizoma Atractylodis Macrocephalae and Radix Salviae Miltiorrhizae, have been reported in literature to alleviate fibrosis by modulating autophagy in animal experiments [20–22]. Moreover, studies have reported that Cortex Mori, Rhizoma Atractylodis Macrocephalae, Rhizoma Phragmitis, Radix Salviae Miltiorrhizae, Radix Platycodi, Rhizoma Pinelliae, and Radix Glycyrrhizae exhibit physiological effects by mediating or triggering autophagy in vitro [23–31]. The relevant molecular targets or autophagy pathways of the herbs included in QFTL reported in the literature are listed in supplementary materials (Supplementary 1). However, the therapeutic use of QFTL and its role in IPF have not been investigated yet.

Therefore, we carried out the present study to evaluate the therapeutic effects of QFTL on pulmonary fibrosis in BLM-induced pulmonary fibrosis rats. This work also revealed the effects of QFTL on inflammation and autophagy in model rats. The results of this study indicated that QFTL had the potential to become a therapeutic drug for IPF in clinical practice.

## 2. Materials and Methods

### 2.1. Materials

The Qingfei Tongluo mixture (125 ml/bottle) was obtained from Tongde Hospital of Zhejiang Province (Hangzhou, China). The Qingfei Tongluo mixture was concentrated and evaporated using a rotary evaporator to obtain 20.24 g/bottle, which was diluted with distilled water when used. Bleomycin was provided by HAN HUI Pharmaceuticals Co., Ltd. (Hangzhou, China). Chloroquine, pyrifenidone, and other common reagents were purchased from Shanghai Aladdin Biochemical Technology Co., Ltd. (Shanghai, China). The hematoxylin and eosin (H&E) staining kit and the Masson staining kit used for tissue pathology staining were purchased from Beijing Solebao Biotechnology Co., Ltd. (Beijing, China). Enzyme-linked immunosorbent assay (ELISA) kits for TGF-*β*, TNF-*α*, and IL-1*β* were purchased from Shanghai Xinyu Biotechnology Co., Ltd. (Shanghai, China). *α*-Smooth muscle actin (*α*-SMA) antibody was purchased from Abcam Shanghai Trading Co., Ltd. (Shanghai, China). The p62 antibody was purchased from Wuhan Servicebio Technology Co., Ltd. (Wuhan, China). The matrix metalloproteinase 12 (MMP12) antibody was purchased from Affinity Biosciences Group Ltd. (Nanjing, China). The mTOR antibody was purchased from Nanjing Bioworld Biotech Co., Ltd. (Nanjing, China).

### 2.2. Experimental Animals and Treatment

**Figure 1:**
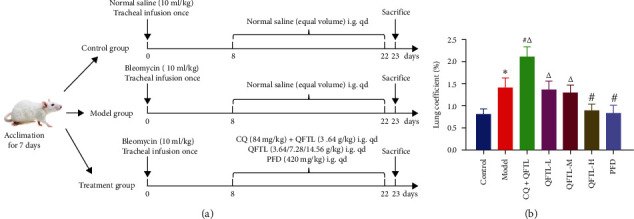
QFTL improves the adverse effects of bleomycin on lung coefficient in rats. (a) An illustration of the timeline for the establishment of the model and delivery of drugs. (b) After 14 days of drug intervention, lung tissue was collected and weighted to calculate the lung coefficient. Data presented are means *±* SD.  ^*∗*^*P* < 0.05 vs. control; ^#^*P* < 0.05 vs. model; ^*Δ*^*P* < 0.05 vs. PFD.

Healthy male SD rats were placed in an SPF environment (22–25°C, 50%–65% humidity, and a 12–12 hr light–dark cycle) and had free access to standard laboratory animal feed and water. Based on the literature and our pre-experimental results [32, 33], we adopted BLM to induce a model of pulmonary fibrosis. After 7 days of acclimation, rats were randomly divided into seven groups (*n* = 8): (1) control group, (2) model group, (3) CQ + QFTL group (treatment with chloroquine at 84 mg/kg and low dose QFTL at 3.64 g/kg), (4) QFTL-L group (treatment with low dose QFTL at 3.64 g/kg), (5) QFTL-M group (treatment with middle dose QFTL at 7.28 g/kg), (6) QFTL-H group (treatment with low dose QFTL at 14.56 g/kg), and (7) PFD group (treatment with pirfenidone at 420 mg/kg). Except for the control group, all other rats were induced pulmonary fibrosis models by tracheal infusion of bleomycin 10 mg/kg. Seven days after BLM injection (Day 8), intragastric administration was started. Each treatment group was administered according to the above protocol, while the normal group and the model group received equal amounts of normal saline. All drugs were administered intragastrically once a day for 14 days. During the experiment, the body weights of the rats were recorded daily. Sixteen hours after the last administration, the rats were weighed, anesthetized with 3% pentobarbital sodium, and sacrificed. Blood samples were drawn from the aortaventralis, while lung tissues were excised and rinsed with 0.9% NaCl solution. An illustration of timeline and workflow is shown in Figure 1(a). All experiments were carried out in accordance with ethical standards guidelines for animal investigations. The Animal Ethics Committee of Zhejiang Academy of Traditional Chinese Medicine approved all protocols on the care of animals and the experiments that were used in this study (Protocol No. 2021-007).

### 2.3. Calculation of Lung Coefficient

Lung coefficient, an indicator of pulmonary fibrosis, was calculated as follows [34]: lung coefficient = (lung weight (g)/body weight (kg)) ×100%.

### 2.4. Enzyme-Linked Immunosorbent Assay

Referring to previous reports [35], the changes in BLM-induced inflammatory factors had similarities in serum, lung tissue, and intestinal tissue. We selected to detect the concentration of inflammatory factors in serum to evaluate the degree of inflammatory response. Each group of rat blood samples was centrifuged at 500 r/min at 4°C for 10 min. The supernatants were analyzed for the concentrations of TGF-*β*1, TNF-*α*, and IL-1*β* with ELISA kits according to the manufacturer's instructions.

### 2.5. HYP Analysis

Hydroxyproline (HYP), a measure of collagen, was used to assess the extent of fibrosis. Lung tissues (0.5 g) were homogenized in a glass tube and the collected supernatant was subjected to subsequent experiments according to the manufacturer's instructions for the HYP assay kit. The level of hydroxyproline was detected by absorbance at 560 nm.

### 2.6. Histological Analysis

After 24 hr of fixation, lung tissue is dehydrated using a fully automatic dehydrator, followed by embedding in paraffin and cutting into 5 *μ*m thick sections. The tissue sample sections were stained with hemoglobin and eosin (H&E) or Masson's trichrome stain to observe inflammatory infiltration and collagen deposition via a light microscope. After image acquisition was completed, the severity of lung fibrosis was semiquantified in tissue sections using the modified Ashcroft method (range of scores 0–8) [36]. Additionally, Image-Pro Plus 6.0 analysis software was used to measure the collagen pixel area and calculate the proportion of positive areas.

### 2.7. Immunohistochemistry of Lung Tissue

The paraffin-embedded lung tissue sections were incubated with 3% H_2_O_2_ for 10 min at room temperature to eliminate endogenous peroxidase activity. After being washed with PBS three times, a high-pressure thermal repair method was used for antigen repair. The sections were then incubated with primary antibodies specific for p62 (1 : 100) and *α*-SMA (1 : 200) overnight at 4°C, followed by incubation with secondary antibody at 30 min at 37°C. After using a DAB kit for color development and counterstained with hematoxylin, the slices were sealed with neutral resin. According to the previous study, the staining results are analyzed using Image-Pro Plus software [37].

### 2.8. Western Blotting

Western blot procedures have been described in previous research [38]. Lung tissue proteins were extracted using radioimmunoprecipitation assay (RIPA) lysis buffer for 30 min on ice. Protein concentrations were determined by the bicinchoninic acid (BCA) protein assay. The harvested proteins were separated on SDS-PAGE and transferred to polyvinylidene difluoride (PVDF) membranes. After blocking in 5% (W/V) nonfat milk for 1 hr, the membranes were incubated with primary antibodies against *α*-SMA (1 : 500), p62 (1 : 1,000), MMP12 (1 : 1,000), mTOR (1 : 1,500), and GAPDH (1 : 500) at 4°C overnight. After washing three times in TBST, the membranes were further incubated with the corresponding secondary antibody (1 : 3,000) at room temperature for 2 hr. Finally, protein band intensities were normalized using GAPDH and quantified with ImageJ software.

### 2.9. Statistical Analysis

GraphPad Prism 8.0 was used for statistical analysis and data are expressed as means ± SD. In multiple-group analysis, one-way or two-way ANOVA with post hoc Tukey's multiple comparison test was used and *P* < 0.05 was considered statistically significant.

## 3. Results

### 3.1. Effect of QFTL on Body Weight and Lung Coefficient

A significant change in body weight (Table 1) and lung coefficient (Figure 1(b)) was observed in the BLM-treated group compared to the control group. As shown in Table 1, after induction by BLM, the weight gain of the rats was relatively slow. After 14 days of QFTL and PFD administration, the weight of the rats showed a rapid growth trend. Changes in lung coefficient also show similar changes. In particular, additional administration of CQ hindered the effectiveness of the QFTL intervention. The weight gain of the rats in the CQ + QFTL group remained slow. Furthermore, compared to the control group and the model group, the lung coefficient of rats in the CQ + QFTL group had a significant increase (*P* < 0.05).

### 3.2. Effect of QFTL on Lung Inflammatory Regulation

**Figure 2:**
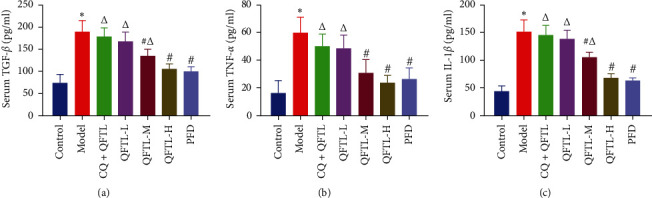
QFTL reduces bleomycin-induced increases in proinflammatory mediators and profibrotic cytokines in rats. Serum concentrations of TGF-*β* (a), TNF-*α* (b), and IL-1*β* (c) in different treatment groups were measured using ELISA kits. TGF-*β*, transforming growth factor-*β*; TNF-*α*, tumor necrosis factor-*α*; IL-1*β*, interleukin-1*β*; data presented are means *±* SD.  ^*∗*^*P* < 0.05 vs. control; ^#^*P* < 0.05 vs. model; ^*Δ*^*P* < 0.05 vs. PFD.

To verify whether QTFL inhibits the inflammatory response in rats with bleomycin-induced pulmonary fibrosis. The levels of TGF-*β*, TNF-*α*, and IL-1*β* were measured (Figure 2). Compared to the control group, higher levels of proinflammatory factors were observed in the model group (*P* < 0.05). After intragastric administration of QFTL and PFD, the levels of proinflammatory factors measured in the serum of each group decreased, especially in the QFTL medium, high dose, and PFD groups, which were significantly lower than the model group (*P* < 0.05). Furthermore, there was no significant difference between the QFTL-H group and the PFD group with the control group.

### 3.3. Effect of QFTL on Lung Histopathology and Fibrosis Development

**Figure 3:**
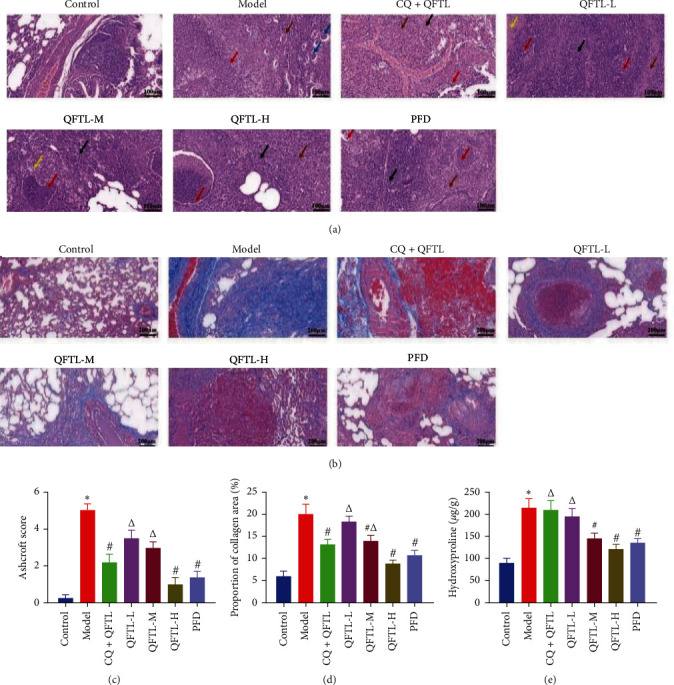
QFTL inhibits bleomycin-induced fibrosis progression. (a) Results of H&E staining showed that QFTL reduced inflammatory damage in rats with bleomycin-induced pulmonary fibrosis. In the control group, the lung tissue structure was normal, with no obvious morphological or pathological changes observed; in the model group, extensive thickening of the alveolar walls was observed in lung tissue, accompanied by a large amount of infiltration of inflammatory cells (black arrow), extensive proliferation of connective tissue (brown arrow), and localized bronchial hyperplasia. The proliferative bronchial lumen was filled with macrophages (blue arrow) and a large number of bronchial epithelial cells were necrotic and shed. A large number of necrotic cell fragments and inflammatory cells (red arrow) were visible in the lumen; in the CQ + QFTL group, extensive thickening of the alveolar walls was observed in lung tissue, accompanied by excessive infiltration of inflammatory cells (black arrow) and a small amount of connective tissue hyperplasia (brown arrow). Bronchial mucosal epithelial cells were necrotic and disappeared, and a large number of inflammatory cells were observed in the lumen (red arrow). In the QFTL-L group, focal connective tissue hyperplasia (brown arrow) can be seen on the alveolar wall in multiple locations of lung tissue, with a significant amount of inflammatory cell infiltration (black arrow). A large number of bronchial mucosal epithelial cells are necrotic and shed, and a large number of necrotic cell fragments (yellow arrow) and inflammatory cells (red arrow) can be seen in the lumen; in the QFTL-M group, focal thickening of the alveolar walls was observed in lung tissue, accompanied by a large amount of infiltration of inflammatory cells (black arrow). A large number of inflammatory cells were often observed in the lumen of the bronchi (red arrow), and local necrosis and disappearance of bronchial epithelial cells were observed (yellow arrow); in the QFTL-H group, a small area of thickening of the alveolar wall was observed in lung tissue, accompanied by a large amount of infiltration of inflammatory cells (black arrow) and a small amount of connective tissue proliferation (brown arrow). There was no obvious shedding of bronchial mucosal epithelial cells, and a large amount of dead debris and inflammatory cells (red arrow) were visible in the lumen of the bronchi. In the PFD group, focal thickening of the alveolar wall was observed in lung tissue, accompanied by a large amount of infiltration of inflammatory cells (black arrow), a small amount of proliferation of connective tissue (brown arrow), a large number of inflammatory cells were observed in the lumen of the bronchi (red arrow), local bronchial proliferation was observed and a small number of inflammatory cells were observed in the proliferative bronchial lumen (red arrow). The arrow indicates the injury area. (b) Representative photomicrographs of rat lung tissues stained with Masson's trichrome. Blue staining indicated collagen deposition. (c) Quantitative assessment of pulmonary fibrosis, as measured by Ashcroft histopathological scoring. After examining the entire section, the mean score of all the fields was taken as the fibrosis score for the section. The higher score indicated the severe fibrosis. (d) Quantitative evaluations of collagen deposition in Massion-stained lung tissue were performed using automated image analysis software (Image-Pro Plus). (e) Quantitative evaluations of hydroxyproline were measured in different treatment groups using HYP assay kit. Data presented are means *±* SD.  ^*∗*^*P* < 0.05 vs. control; ^#^*P* < 0.05 vs. model; ^*Δ*^*P* < 0.05 vs. PFD.

As depicted in Figure 3(a), the model rats showed a significant progression of pulmonary fibrosis, characterized by extensive thickening of the alveolar walls, accompanied by large amounts of inflammatory cell infiltration and proliferation of connective tissue. However, dose-related improvements were observed in the lung histopathology of rats in the QFTL intervention group. In particular, similar lung structural damage was observed in lung tissue of rats in the CQ + QFTL group compared to the model group. Quantitative evidence for the evaluation of fibrosis using the Ashcroft score is shown in Figure 3(c). Subsequently, a quantitative evaluation of collagen deposition in lung tissue was carried out by Masson staining (Figures 3(b) and 3(d)) and similar results were presented. HYP analysis (Figure 3(e)) also showed similar results.

### 3.4. Effect of QFTL on the Expression of Pulmonary Fibrosis-Related Proteins

**Figure 4:**
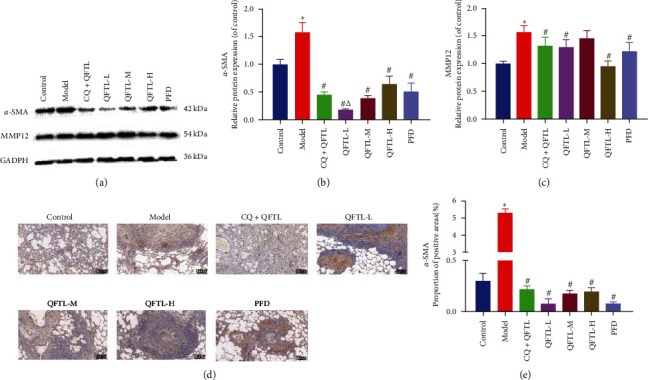
QFTL attenuates bleomycin-induced fibroblast activation. (a) Western blot detection shows the expression of pulmonary fibrosis-related proteins *α*-SMA and MMP12. Results are expressed as relative ratios of band density of *α*-SMA (b) and MMP12 (c). (d) Immunohistochemical staining of *α*-SMA was performed for further validation. Representative photomicrographs of immunohistochemical staining in lung tissues from different treatment groups. (e) Quantitative evaluations were performed by mage-Pro Plus 6.0 software to calculate the positive area proportion of *α*-SMA. *α*-SMA, *α*-smooth muscle actin; MMP12, matrix metalloproteinase 12; data presented are means *±* SD.  ^*∗*^*P* < 0.05 vs. control; ^#^*P* < 0.05 vs. model; ^*Δ*^*P* < 0.05 vs. PFD.

In order to confirm the influence of QFTL in BLM-Induced pulmonary fibrosis. Protein expressions of *α*-SMA and MMP12 were determined by Western blot and immunohistochemistry.  Figure 4(a)–4(c) (Supplementary 2) showed that QFTL significantly downregulated the protein expression levels of *α*-SMA and MMP12 in BLM-induced pulmonary fibrosis rats (*P* < 0.05). Similarly, the immunofluorescence assay revealed that QFTL could reduce the BLM-induced expression level of *α*-SMA in rats (Figures 4(d) and 4(e)). When comparing the expression levels of *α*-SMA protein between the CQ + QFTL group and the QFTL group, we noticed a visible difference in the values between the two groups, and further statistical results showed significant differences (*P* < 0.05).

### 3.5. Effect of QFTL on the Expression of Autophagy-Related Proteins

**Figure 5:**
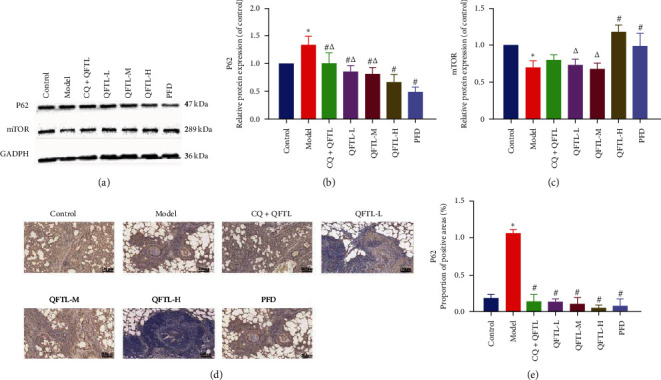
QFTL promotes fibroblast autophagy in bleomycin-induced pulmonary fibrosis. (a) Western blot detection shows the expression of pulmonary autophagy-related proteins p62 and mTOR. Results are expressed as relative ratios of band density of p62 (b) and mTOR (c). (d) Immunohistochemical staining of p62 was performed for further validation. Representative photomicrographs of immunohistochemical staining in lung tissues from different treatment groups. (e) Quantitative evaluations were performed by mage-Pro Plus 6.0 software to calculate the positive area proportion of p62. mTOR, mechanistic target of rapamycin; data presented are means *±* SD.  ^*∗*^*P* < 0.05 vs. control; ^#^*P* < 0.05 vs. model; ^*Δ*^*P* < 0.05 vs. PFD.

To explore whether QFTL improved pulmonary fibrosis through autophagy, the expression of autophagy-related proteins p62 and mTOR in fibrotic lung tissues was measured. As shown in Figure 5(a)–5(c), (Supplementary 2), rats in the model exhibited significantly higher protein expression of p62 (*P* < 0.05), as well as markedly decreased protein expression of mTOR (*P* < 0.05) compared to the control group. As expected, after the QFTL and PFD intervention, the trend of changes in the expression of the aforementioned proteins was attenuated. Similarly, immunohistochemistry techniques were once again used to verify the effect of QFTL on the increase in BLM-induced protein expression of p62 (Figures 5(d) and 5(e)). Quantitative evaluation showed that, compared to the model group, therapeutic drugs significantly reduced the expression of p62 (*P* < 0.05), and there were no significant differences between the treatment groups (*P* > 0.05).

## 4. Discussion

IPF is a chronic and progressive interstitial lung disease with a high incidence rate and mortality. Although nintedanib and pirfenidone have been approved by the FDA as drug therapies for IPF, it is indisputable that lung transplantation remains the only treatment method to improve prognosis. In recent years, COVID-19 has swept the world. Relevant epidemiological data showed that the incidence of fibrosis after COVID-19 will become apparent with time, and even patients discharged from rehabilitation still exhibit a high rate of abnormalities in fibrotic lung function [39]. Given that COVID-19-induced pulmonary fibrosis and IPF have similar risk factors and biological processes [40]. We assumed that QFTL had the potential to treat pulmonary fibrosis and tried to confirm it in this study.

The bleomycin model is the best characterized animal model available for preclinical pulmonary fibrosis experiments, as recommended by the American Thoracic Association [41]. The bleomycin delivery routes reported in the literature include multiple routes of administration, including tail vein injection, tracheal perfusion, and intraperitoneal injection. When considering bleomycin, most investigators use a single intratracheal administration [42]. This is because repeated intratracheal administrations [43] or repeated intraperitoneal administrations [44] may offer more robust and nonresolving fibrotic pathology. Therefore, we chose a single intratracheal injection of bleomycin to induce a pulmonary fibrosis model. The bleomycin model is based on the initiation of inflammatory processes (Days 0–7) and can observe the formation of pulmonary fibrosis after 14 days, with a plateau phase for the next 2 weeks (Days 14–28) [45]. The consensus of scholars is that it is more meaningful to administer the drug therapeutically after reaching its peak during the acute inflammatory phase of the lung injury response and presenting evidence of fibrosis (e.g., 7−10 days after bleomycin instillation) [41]. The specific timing of therapeutic drug intervention may be influenced by animal strain, dose, and method of bleomycin administration. The induction dose of bleomycin varies depending on the animal species, and the commonly used dose reported in the literature for rats is 5–10 mg/kg [46, 47]. Referring to the dose–response effect of bleomycin on inflammation and fibrosis [48], we attempted to induce a fibrosis model by infusion of 10 mg/kg bleomycin in the pre-experiment. On the 7th day of bleomycin induction, evidence of early fibrosis could already be found in pathological histological sections. In summary, we decided to carry out the work according to the experimental steps mentioned above.

As lung tissue lesions can lead to cellular edema, overflow of inflammatory substances, and congestion of capillaries, ultimately leading to an increase in the lung coefficient. Lung tissue lesions and organ coefficient are considered intuitive indicators of lung consolidation [49]. In our study, QFTL could reduce the lung coefficient caused by bleomycin-induced pulmonary fibrosis in rats without affecting normal growth. The early pathological features of pulmonary fibrosis are mainly diffuse alveolar inflammation. A variety of inflammatory cells and cytokines stimulate fibroblasts to proliferate excessively and transform into myofibroblasts, resulting in abnormalities in the metabolism of the ECM [50]. H&E staining can show that QFTL can improve inflammatory lesions in rat lung tissue, including altering cell necrosis, shedding, and reducing inflammatory cell infiltration. Furthermore, the hydroxyproline content in lung tissue is commonly used as a gold indicator to evaluate the collagen content [51]. *α*-SMA is a typical marker of the proliferation and differentiation of lung fibroblasts into myofibroblasts after various pathogenic factors affect lung tissue [52]. MMP12 plays a key role in regulating inflammation regression and is highly expressed in tissues rich in elastic fibers in the lungs [53]. Consistent with the lung coefficient and histopathological experimental results, QFTL could significantly improve the proportion of fiber fibers in lung tissue, reduce collagen fiber generation, and reduce the expression of mmp12, *α*-SMA, and the content of hydroxyproline in lung tissue. All of these results showed that QFTL could significantly alleviate BLM-induced pulmonary fibrosis in rats.

Although the pathogenesis of pulmonary fibrosis is still unclear, increasing evidence confirms that inflammatory reactions play a complex and crucial role in it. Existing research has confirmed that the characteristic of pulmonary fibrosis is the loss of endothelial function and subsequent activation of the immune system, which is considered a direct result of the influence of intracellular proinflammatory responses [54]. Numerous studies on pulmonary fibrosis suggest that various proinflammatory cytokines and cell markers are involved in the development of this pathology, and their increased expression is associated with a more intense fibrosing process [55]. Typical cytokines cited and extensively discussed include TGF-*β* [56], TNF-*α* [57], and IL-1*β* [58]. In the current study, we observed an upregulation of these cytokines in the serum of BLM-induced rats, which is consistent with previous reports. Furthermore, QFTL can significantly reduce the content of inflammatory factors in rats with pulmonary fibrosis, while improving pulmonary fibrosis. Taking into account the connection between the inflammatory response and pulmonary fibrosis, the role of QFTL is self-evident.

Autophagy is a highly conserved and genetically controlled pathway in eukaryotic cells, which transports damaged, denatured or aged proteins, and organelles to lysosomes for digestion and degradation to achieve cell recycling and reuse. Autophagy is regulated by multiple signaling pathways and its process can be roughly divided into four consecutive steps: autophagy induction, autophagosome formation, degradation, and reuse [59, 60]. Among them, mTOR is a master regulatory molecule in the autophagy induction stage, which is positively regulated by the Akt and MAPK signaling pathways, while negatively regulated by the Akt and MAPK signaling pathways. Increased expression enhances the ability to regulate autophagy under stress [13]. During the formation of autophagosomes, p62 can serve as a measurement indicator of autophagic flow, as blocking autophagy can lead to accumulation of P62. The decrease in its expression is a response to increased autophagic activity [61]. In this study, immunohistochemistry and Western blot results showed that QFTL could significantly increase the expression of mTOR protein in bleomycin-induced pulmonary fibrosis in rats, while reducing the expression of the p62 protein. This demonstrated the effect of QFTL on autophagy of pulmonary fibrosis. What interests us is that when CQ was added, similar results were obtained. This may be because the chloroquine regulatory pathway is located downstream of signaling pathways related to mTOR and P62. This indicated that further research is needed to fully reflect the specific mechanism of QFTL in autophagy.

Sufficient evidence has confirmed that autophagy plays a protective role in the progression of pulmonary fibrosis. Autophagy is involved in the activation of lung fibroblasts and interstitial transformation of human pulmonary bronchial epithelial cells, regulating the secretion of various cytokines, and promoting and inhibiting the development of fibrosis by regulating oxidative stress in the process of pulmonary fibrosis [8]. Published research has found that traditional Chinese medicine, such as Ligustrazin [62], Fu–Zheng–Tong–Luo formula [63], Number 2 Feibi recipe [64], can reduce oxidative stress in lung tissue, promote autophagy, and therefore slow or inhibit the degree of pulmonary fibrosis. As mentioned above, compared to the model group, rats in the QFTL-L group showed a significant decrease in HYP levels (Figure 3(e)) and *α*-SMA protein expression (Figures 4(b), 4(d), and 4(e)), but the addition of CQ blocked the trend of improvement. There was a significant difference between the two groups (*P* < 0.05). Similar reversal phenomena could also be observed in histopathological analysis. These results indicated that QFTL attenuates pulmonary fibrosis through multiple pathways, but autophagy was clearly involved. Unfortunately, the difference in the proportion of collagen area and MMP12 protein expression between the two groups was not statistically significant. We speculated that it is related to an insufficient dose of QFTL. On the basis of the same factors, we were unable to obtain evidence of the exact causal relationship between BLM-induced inflammation and autophagy. However, we have observed the potential of QFTL to improve pulmonary fibrosis through mediated autophagy, and further confirmation research will be conducted in the future.

## 5. Conclusions

Our findings revealed that QFTL can effectively attenuate bleomycin-induced inflammation and pulmonary fibrosis in rats. Mechanisms were involved in promoting autophagy.

## Figures and Tables

**Table 1:** Body weight of rats in different groups.

	Control	Model	CQ + QFTL	QFTL-L	QFTL-M	QFTL-H	PFD
Day 1	234.32 ± 9.16	236.32 ± 8.11	233.80 ± 9.62	228.04 ± 6.11	229.26 ± 6.11	237.42 ± 8.11	233.80 ± 8.62
Day 7	243.40 ± 11.90	245.28 ± 10.62	247.80 ± 10.62	239.58 ± 7.62	239.36 ± 7.62	238.80 ± 10.62	245.80 ± 10.62
Day 14	261.33 ± 14.59	263.04 ± 12.58	269.24 ± 12.58	264.72 ± 11.58	266.32 ± 11.58	271.24 ± 12.58	267.24 ± 12.58
Day 17	289.74 ± 19.24	265.80 ± 14.24	265.68 ± 14.24	272.92 ± 13.24	279.48 ± 13.24	280.68 ± 14.24	280.68 ± 14.24
Day 20	291.55 ± 18.74	271.88 ± 17.74	257.92 ± 17.74 ^*∗*^	289.10 ± 17.74	292.64 ± 17.74	297.92 ± 17.74	287.92 ± 17.74
Day 23	312.80 ± 18.91	281.66 ± 16.91 ^*∗*^	300.28 ± 12.91	294.42 ± 12.91	308.56 ± 9.91	310.28 ± 12.91	315.28 ± 12.91
Day 26	339.35 ± 19.90	285.50 ± 17.90 ^*∗*^	309.56 ± 17.90	311.26 ± 9.90	320.06 ± 12.90^#^	331.56 ± 17.90^#^	329.56 ± 17.90^#^
Day 28	343.35 ± 19.90	295.50 ± 17.90 ^*∗*^	313.56 ± 17.90	321.26 ± 9.90	330.06 ± 12.90^#^	341.56 ± 17.90^#^	339.56 ± 17.90^#^

*Note*.  ^*∗*^*P* < 0.05 vs. control; ^**#**^*P* < 0.05 vs. model.

## Data Availability

All data are available from the corresponding authors upon reasonable conditions.

## Supplementary Materials

Supplementary 1 Literature related to autophagy on herbs in QFTL.

Supplementary 2 The protein expression levels of *α*-SMA, MMP12, P62, and mTOR in different treatment groups were determined by Western blotting.
