# Supplementary material for: Qingfei Tongluo Mixture Attenuates Bleomycin-Induced Pulmonary Inflammation and Fibrosis through mTOR-Dependent Autophagy in Rats
Source: Mediators Inflamm. 2024 Feb 8;2024:5573353. doi: 10.1155/2024/5573353 (PMC10869187; doi:10.1155/2024/5573353)
Supplement: Supplementary 1 — Literature related to autophagy on herbs in QFTL. [file 5573353.f1.docx]

Table S1: Literature related to autophagy on herbs in QFTL

| Herbs | Animal/Cell line | Molecular targets /Pathways | Phenotype/Disease |
| --- | --- | --- | --- |
| Rhizoma Atractylodis Macrocephalae | C57BL/6 mice | LC3-II/I | Hepatic fibrosis [20] |
|  | interstitial cells of Cajal | PI3K/Akt/mTOR | Constipation [23] |
| Radix Salviae Miltiorrhizae | Sprague-Dawley rats, LX‐2 | LC3-II/I, p62 | Hepatic fibrosis [21] |
|  | Sprague-Dawley rats, LX‐2 | LC3-II/I, p62 | Renal fibrosis [22] |
|  | SW620 Ad300 | ROS/p38 MAPK/NF-κB | Drug resistance [24] |
|  | A549 | ROS/JNK | Apoptosis [25] |
|  | NB4 | PI3K/Akt/mTOR | Apoptosis [26] |
| Cortex Mori | NCI-H460 | AMPK/mTOR/AKT | Apoptosis [27] |
| Rhizoma Phragmitis | *A.castellanii* trophozoites | AcAtg3, 8b, 12, 16 | Apoptosis [28] |
| Radix Platycodi | A549 | AMPK/mTOR/AKT, MAPK | Apoptosis [29] |
| Rhizoma Pinelliae | TPC‐1, BCPAP | MAPK | Apoptosis [30] |
| Radix Glycyrrhizae | SH‑SY5Y | LC3-II/I, Beclin-1 | Parkinsonism [31] |
