# Supplementary material for: Qingfei Tongluo Mixture Attenuates Bleomycin-Induced Pulmonary Inflammation and Fibrosis through mTOR-Dependent Autophagy in Rats
Source: Mediators Inflamm. 2024 Feb 8;2024:5573353. doi: 10.1155/2024/5573353 (PMC10869187; doi:10.1155/2024/5573353)
Supplement: Supplementary 2 — The protein expression levels of α-SMA, MMP12, P62, and mTOR in different treatment groups were determined by Western blotting. [file 5573353.f2.docx]

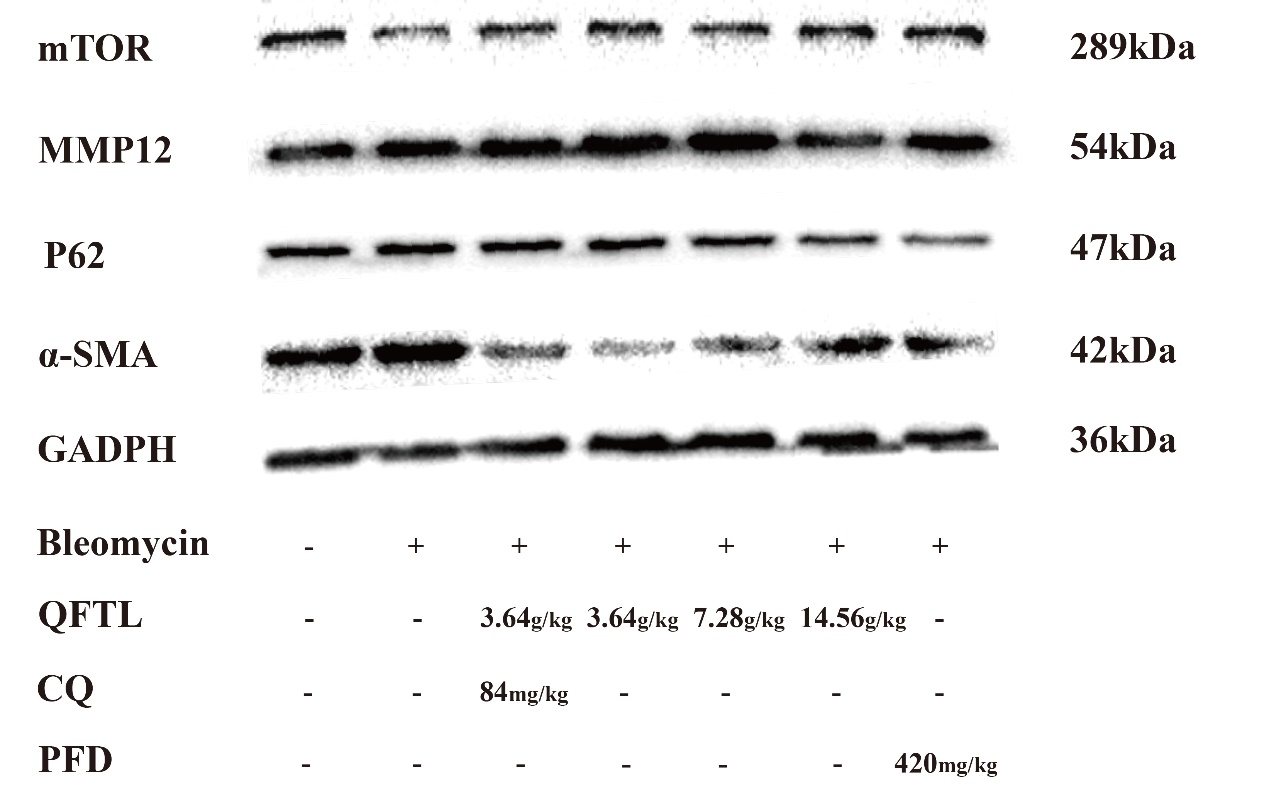


Figure S1: The protein expression levels of α-SMA, MMP12, P62 and mTOR in different treatment groups were determined by western blotting. α-SMA: α-smooth muscle actin; MMP12: matrix metalloproteinase 12; mTOR: mechanistic target of rapamycin.
